# *Mare-MAGE* curated reference database of fish mitochondrial genes

**DOI:** 10.1186/s12863-023-01119-4

**Published:** 2023-03-17

**Authors:** Yassine Kasmi, Erik Eschbach, Reinhold Hanel

**Affiliations:** Thünen Institute of Fisheries Ecology, Herwigstraße 31, 27572 Bremerhaven, Germany

**Keywords:** Mare-MAGE, Mitochondrial database, Fish, Metabarcoding, eDNA

## Abstract

Biodiversity assessment approaches based on molecular biology techniques such as metabarcoding, RAD-seq, or SnaPshot sequencing are increasingly applied in assessing marine and aquatic ecosystems. Here we present a new reference database for fish meta-barcoding based on mitochondrial genes. The ***Mare-MAGE*** database contains quality-checked sequences of the mitochondrial 12S ribosomal RNA and Cytochrome c Oxidase I gene. All sequences were obtained from the National Center for Biotechnology Information- GenBank (NBCI-GenBank), the European Nucleotide Archive (ENA), AquaGene Database and BOLD database, and have undergone intensive processing. They were checked for false annotations and non-target anomalies, according to the Integrated Taxonomic Information System (ITIS) and FishBase. The dataset is compiled in ARB-Home, FASTA and Qiime2 formats, and is publicly available from the ***Mare-MAGE*** database website (http://mare-mage.weebly.com/). It includes altogether 231,333 COI and 12S rRNA gene sequences of fish, covering 19,506 species of 4,058 genera and 586 families.

**Supplementary Information:**

The online version contains supplementary material available at 10.1186/s12863-023-01119-4.

## Background

Monitoring and surveillance of marine biodiversity is crucial for ecosystem conservation since it can provide decision makers appropriate arguments for the preservation and sustainable use of natural resources. Conventional biodiversity assessment tools are limited in terms of flexibility and costs, and they can be subject to bias and uncertainty [1, 2]. Thus, new molecular genetics-based approaches such as Metabarcoding, Restriction site-associated DNA sequencing (RADseq) markers, SNaPshot sequencing and Quantitative Polymerase Chain Reaction (qPCR) are increasingly used as qualitative and semi-quantitative methods, especially in the marine studies domain [3, 4].

**Fig. 1:**
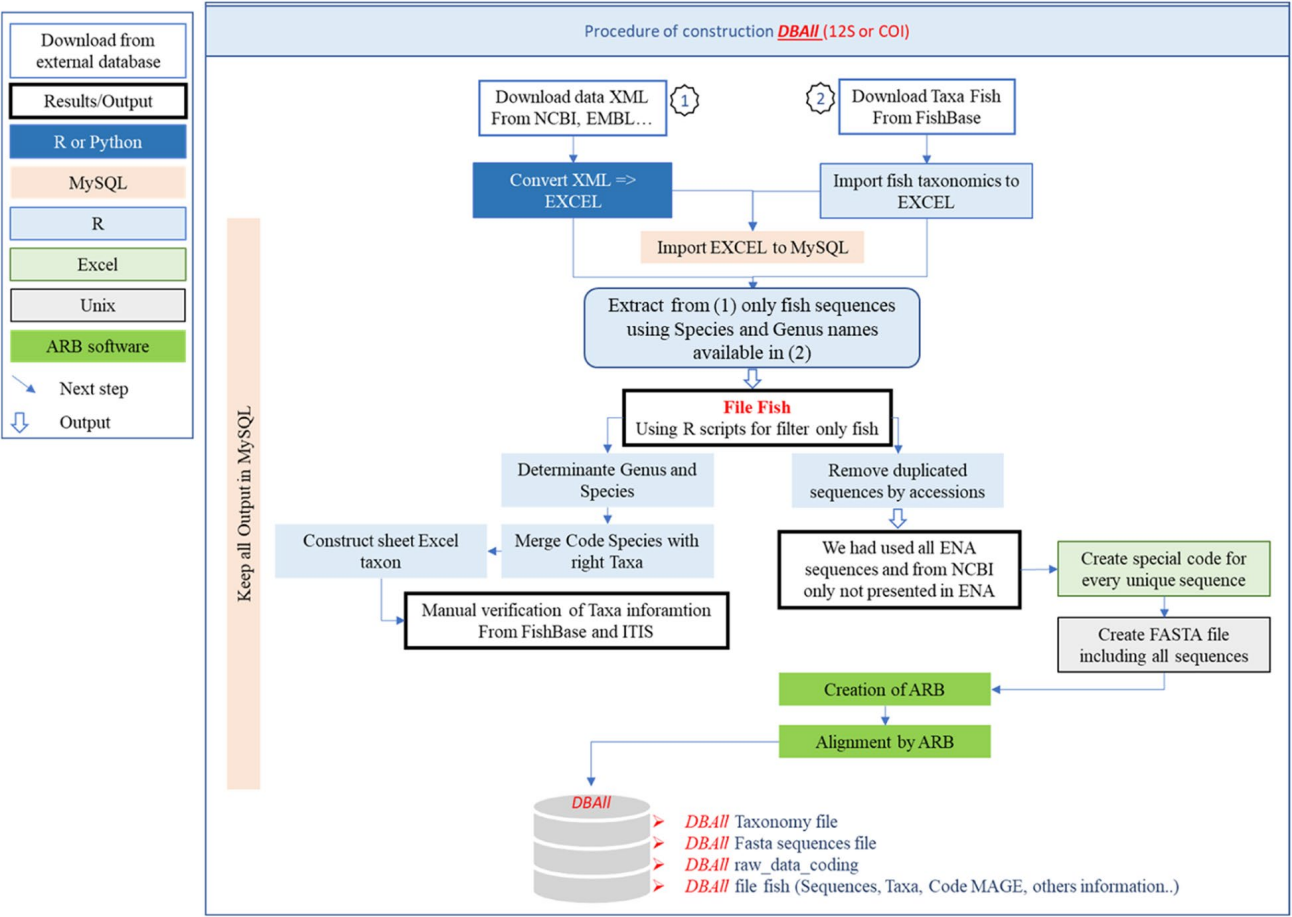
Detailed schematic view of the ***Mare-MAGE-DBAll*** development process. The process mentioned here is the same for the both COI and 12S genes. The sub-database "Mare-MAGE-*DBAll*" contains raw-data collected from various public databases (ENA, AquaGene, GenBank, NCBI, BOL), consisting of all fish derived 12S or COI sequences available without any correction or validation, except for some taxonomic revisions according to the new nomenclature published by ITIS and FishBase. The MS Excel file “Other-Info” includes all sequence information (length, taxonomy and remarks) together with the relevant code in ***Mare-MAGE***, the source of the database (NCBI, BOLD,..) and the author’s name for the original data

All living organism shed DNA in their environment, and it is possible to collect this DNA, amplify it via PCR using primer sets targeting DNA marker regions and sequence the resulting amplicons using NGS [5–7]. While the use of environmental DNA (eDNA) is a manageable and almost “routine” method in microbiology, it remains a challenge in marine biology and ichthyodiversity monitoring, despite first publications dating back to 2011 (Fig. 1). Such studies face various challenges with respect to bioinformatic analyses [8, 9]. Among these challenges are: (1) The need for a fairly good knowledge in the field of programming and coding, since almost all analysis platforms operate in a Unix environment. Some pipelines have proven to be very useful in microbiological eDNA-metagenomics and meta-taxonomics, with easier interfaces for a larger user community. These pipelines are less suited to eDNA studies, due to (2) a lack of availability of a comprehensive reference database covering a sufficient number of species and (3) the incompatibility with analytical tools. The first paper was published in 2011 and since then more than 300 articles have been published and indexed in Scopus. Compson et al. [10] in their systematic review article showed that the number of articles and papers published on eDNA on various organisms and in multiple domains has been increasing significantly during the last 5 years. The authors also found that the study of eDNA is no longer just an academic research topic, but is beginning to be touched by industry and decision makers, which explains its importance and dynamics [10].

For eDNA studies, many mitochondrial genes have been used, such as the 16S small subunit ribosomal RNA gene (16S rRNA), the 12S small subunit ribosomal RNA gene (12S rRNA), as well as the Cytochrome c Oxidase I (COI) and Cytochrome b (MT-CYB) genes. Like all mitochondrial genes, the 12S rRNA and COI genes are present in many more copies per cell compared to nuclear DNA regions. This high frequency in the number of mitochondrial sequences per cell facilitates detection and lowers the detection limit even of low-abundance species. The mitochondrial 12S rRNA is one of the ribosomal RNAs encoded by the mitochondrial genome of eukaryotes, a component of the small subunit of the mitochondrial ribosome and of particular interest in phylogenetic studies [11, 12]. COI encodes subunit I of the mitochondrial Cytochrome c Oxidase, and is involved in the respiratory chain of mitochondria. Moreover, these genes have a particularly high sequence variability for the distinction between species, which predestined them for the use in the barcode of life project [13].

To our knowledge, there is currently no global (worldwide), specific, quality checked and taxonomy-revised 12S rRNA database for fish. The most commonly used COI database for fish identification is BOLD Systems [13], which includes FISH-iBOL [14] as a sub-database for fish. However, in spite of the number of species covered, the BOLD Systems database requires complicated pre-processing before being compatible with many common bioinformatics pipelines (Obitools, Qiime2, vsearch, dada2), limiting its use in eDNA studies. 16S and 18S rRNA genes are represented in the Silva database [15], which also includes 23S rRNA gene information. While the Silva database is compatible with most pipelines, it is more focused on microbiota and Archaea, with very limited information on eukaryote taxa and almost none on fish. MitoFish database is an original NCBI database that essentially contains about 2800 complete mitochondrial sequences of about 600 fish species, covering only 1.7% of all known fish species (35,100 species according to Fishbase [23]) [16]. FishCARD database is a new 12S gene database specific for the coast of California and it covers 600 species [17].

In this paper, we present the Mare-Marine-Gene (***Mare-MAGE***) database as a comprehensive quality controlled and taxonomically reviewed reference database for 12S rRNA and COI sequences of fish to be ready for use by convenient analysis pipelines.

## Construction and content

### Data sources and content

The ***Mare-MAGE*** database contains mitochondrial 12S rRNA and COI gene sequences of fish, collected from The National Center for Biotechnology Information-GenBank (NCBI) [18], AquaGene [19], BOLD [13] and the European Nucleotide Archive (ENA) [20] databases as of January 21^th^, 2022 for the 12S rRNA gene and January 26^th^, 2022 for the COI gene. NCBI and ENA data were downloaded as XML (Extensible Markup Language) files, using the following keywords: “12S RNA”, “12 s RNA”, “12S ribosomal RNA”, “12 s rRNA” and “12 s” for 12S rRNA gene, and “COI RNA”, “COI”, “CO1”, “CO1”, “MT-CO1”, “COX1” and “Cytochrome c oxidase I” for COI gene. We used the filter “species = animal” in GenBank database. The XML files were imported and transformed into tables via the Data-XML function in Microsoft Excel. The data from AquaGene [19], BOLD [21] databases were downloaded as Excel files.

### Data processing

All sequences were initially filtered by their accession number to eliminate duplicates in the same file via R and Excel, then revised according to their accessions to keep only those sequences annotated by the authors as 12 s rRNA or COI. A second filter step to remove duplicates between GenBank and ENA was performed via an R script. A fish taxonomy file of all ranks (class, order, family, genus and species) was obtained from FishBase and included 35,100 species [22, 23]. The fish taxonomy file was used as reference list of known fish species. Using a custom R-script and the fish taxonomy file as a reference, all data were filtered to include only accessions where the original authors identified the sequence as originating from a distinct fish species.

Each accession received a new identification code in the ***Mare-MAGE Database***, 12sDBxxxx for 12 s rRNA and CO1DBxxx for COI genes. The original accessions with all information and the new codes in the ***Mare-MAGE database*** were saved in a table in MySQL, which includes the following fields: the original accession numbers, the new code in ***Mare-MAGE***, the original database, and the original genus and species names published by the original authors (Fig. 1). All sequences and related data extracted from GenBank, AquaGene, BOLD and ENA databases have been stored locally in a relational database system (MySQL). The sequences were saved in FASTA format with the new ***Mare-MAGE*** codes, and formed the seed for a new ARB-home database consisting of aligned and unaligned sub-databases.

### Curation of taxonomy

Each sequence in the ***Mare-MAGE-DB*** has the taxonomy assignments of either AquaGene, BOLD, ENA or GenBank databases, depending on its origin. The taxonomy (genus and species) was retrieved together with the sequence in the XML files. Subsequently, all organism names were synchronized and manually reviewed to revise, correct and complete the sequence taxonomy based on information provided by The Integrated Taxonomic Information System (ITIS) [24] and FishBase [22, 23] to remain consistent with the most up to date scientific names according to published recommendations, i.e. domain, class, order and family. All former names and taxa are stored in the database, as well as in the corresponding field in the ARB databases.

### Quality filtration and rating

All sequences underwent a global alignment by MAFFT to confirm the 12S and COI gene region. Subsequently each family underwent an alignment by ARB-home, aiming at finding ambiguities in the sequences and the global relationship via a distance matrix generated by ARB-home [25]. The data of each family were uploaded to the ARB-home environment, to build a database, and standardized by aligning the unique sequences for each family. Finally, a *Sq* score—“Sequences Quality score”—was calculated according to Eq. 1, as published by Pruesse et al. [15].

Sequences with a *Sq* criterion < 30% were rejected, as mentioned by Pruesse et al. [15]. Since the quality of the final datasets is critically dependent on the quality of the sequence alignments, the datasets were checked during the database construction. In this process, all sequences that could not be unambiguously aligned were removed from the database.

1 $$Sq=1-\left(\frac{\left(\frac{A}{{A}_{max}}\right)+\left(\frac{H}{{H}_{max}}\right)+\left(\frac{V}{{V}_{max}}\right)}{3}\right)*100,$$

where *Sq* corresponds to sequence quality score, A to ambiguities, H to homopolymer and V to vector identity. A second global alignment of all sequences took place to build the phylogenetic tree. Conspecific sequences from the same genus were kept only at phylogenetically very close distance. The phylogenetic trees were examined at the level of each genus and family; if the sequences were as expected, they were retained, if not, the discrepancies were examined.

The ARB alignment is based on two different parameters (consensus and by reference) using **zebrafish** (*Danio rerio*) reference sequences (accession numbers KM244705 and NC_002333), followed by a global phylogenetic tree produced for all species using the “neighbor joining algorithm” NJ incorporated in the ARB program.

### Database composition

**Fig. 2:**
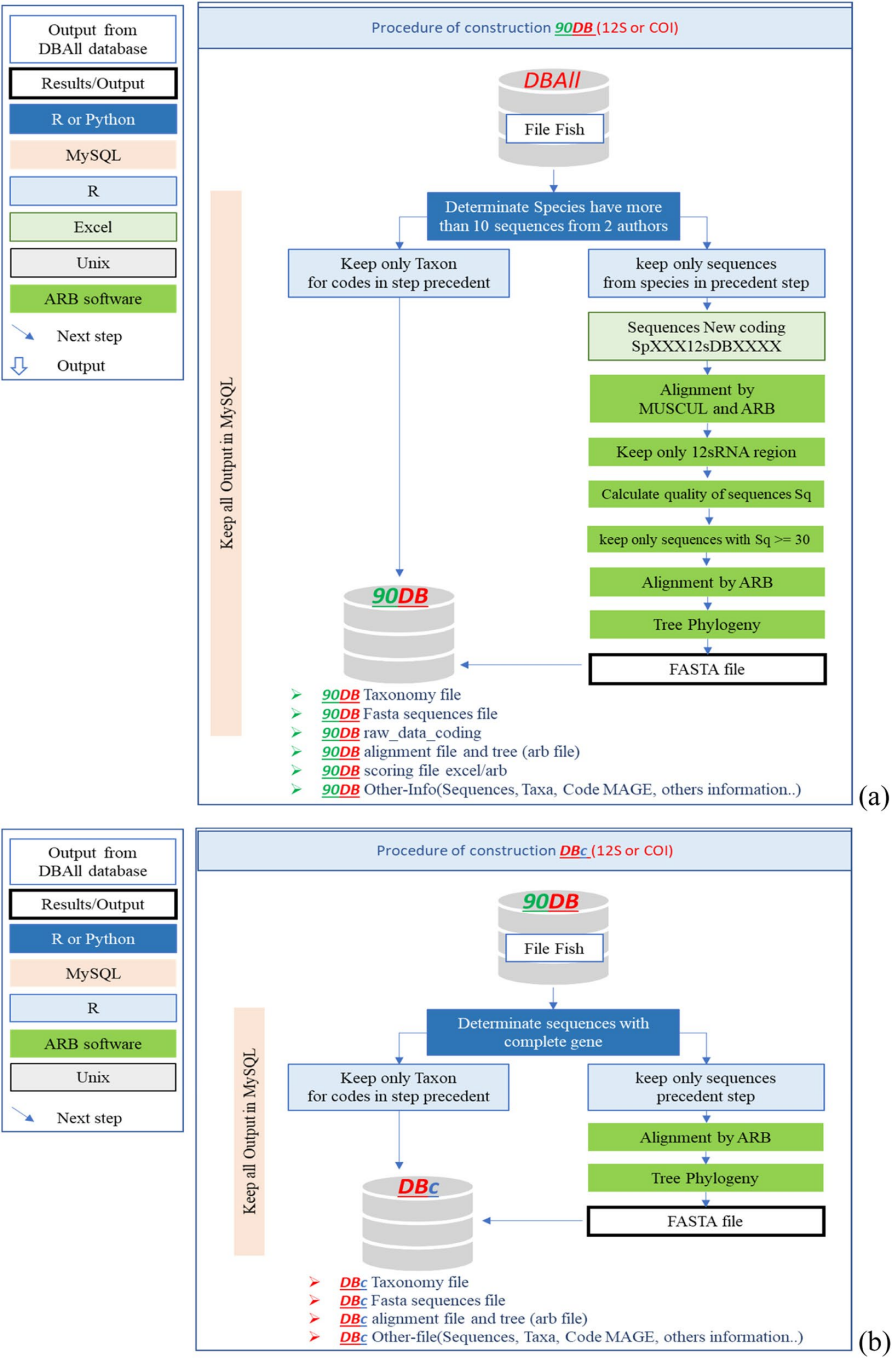
Description of the procedure followed during the development of Mare-MAGE-90DB (**a**), the complete gene sub-database Mare-MAGE-DBc (**b**) and the filtering of sequences. This process is identical for both genes

The ***Mare-MAGE database*** includes 3 sub-databases for each gene:

- ***Mare-MAGE-DBAll***: includes all fish-specific 12S or COI sequences available in the NCBI and ENA databases, without further cleaning or validation, except for a nomenclature revision (Fig. 1). For each gene we provide a sub-database ***Mare-MAGE-DBAll*** with taxa files, FASTA files, and ARB files for each gene sub-database. In addition, we provide the dada2 and Qiime2 format for each gene on our website [26].
- ***Mare-MAGE-90DB*** includes only entries of species represented by more than 10 individual sequences with a similarity higher than 98%. ***Mare-MAGE-90DB*** is derived from ***Mare-MAGE-DBAll***. It was processed and filtered by organism name and sequence frequency via an R script. Only sequences belonging to taxa with at least 10 sequences from 2 different authors in the ***Mare-MAGE-DBAll*** were retained. Every family then underwent an alignment by ARB to produce a similarity matrix. By combining the output of the similarity matrix and the Sq score calculated by ARB, the sequences with high similarity were selected as recommended by Pruesse et al. [15] (Fig. 2a). For each gene we provide a sub-database ***Mare-MAGE-90DB*** with a taxa file, a FASTA file, and an ARB file for each gene sub-database. The other files are available by request from the corresponding author.
- ***Mare-MAGE-DBc*** is a sub-database containing only the sequences that cover the complete 12S rRNA and COI genes in ***Mare-MAGE-90DB***. In order to construct this sub-database, we created a seed for each family from the data issued from complete mitochondrial genome sequences published in NCBI, before aligning all sequences of ***Mare-MAGE-90DB*** from this seed. Only the sequences that cover the whole 12S rRNA or COI gene were collected. Subsequently, ***Mare-MAGE-DBc*** was processed and filtered by the name of the species and the number of sequences representing it via an R script (Fig. 2b). For each gene we provide a sub-database ***Mare-MAGE-DBc*** with a taxa file, a FASTA file, and an ARB file for each gene sub-database. The other files are available by request from the corresponding author.

***Mare-MAGE-DBAll*** includes all collected sequence information, 92–18,000 bp and 100–17 000 bp for 12S rRNA and COI genes, respectively. The large size span is due to the presence of whole mitochondrial genomes together with only partial gene sequences as applied by many researchers. Complete gene sizes of 12S rRNA and COI are around 1000 bp and 1500 bp, respectively. Since ***Mare-MAGE-DBAll*** contains only the raw data, single gene sequences were not extracted from the whole mitochondrial genomes.

To be present in ***Mare-MAGE-90DB*** sequences require minimum lengths of 100 bp and 500 bp for 12S and COI genes, respectively. However, for ***Mare-MAGE-DBAll*** only sequences longer than 900 bp were kept.

### Database validation

Taxonomic assignment is a crucial step in each eDNA analysis. Thus, the choice of the reference database may affect post-analyses and subsequent interpretation of the results. Therefore, we performed a comparison between the output of ***Mare-MAGE*** and NCBI with regard to taxonomic assignment, in order to evaluate the performance and demonstrate the utility of the ***Mare-MAGE*** database and its three sub-databases.

To accomplish this task for COI, we used a raw data set published by Günther et al. [27] as the outcome of an eDNA study on North Sea water samples based on universal COI primers (SRA accession: SRR7661161, Project accession: PRJNA485040). For the 12S rRNA gene, a seawater sample (5L) was collected during a from the North Sea fisheries research survey on board FV Walther Herwig III (mission number WH428) from 9 July to 6 August 2019. Immediately after sampling, the water was filtered through a 0.45 µM membrane and stored at -20 °C, until DNA extraction within 4 weeks. The eDNA extraction was performed via the Phenol–chloroform method, followed by centrifugal column purification with Monarch™ PCR & DNA Cleanup Kit (New England Biolabs) in a dedicated room at the genetics laboratory of the Thünen Institute of Fisheries Ecology, Bremerhaven. MiSeq (NGS) library preparation and sequencing were performed according to the protocol published by Miya [28] using MiFish primers targeting the 12 s rRNA gene (the Fastq file is available in *figshare* [26]-validation data/12S-).

For taxonomic assignment, the Vsearch pipeline was used as the reference pipeline of NCBI followed by BLASTN. The Fastq files initially underwent a treatment by Vsearch to assemble the forward and reverse sequences, then the primers and the adaptors were removed by Cutadapt (version 3.4). Subsequently, the Vsearch pipeline was used for the filtering the steps, to remove duplicates and cluster the OTUs. Thereafter, the BLASTN pipeline was used to assign the OTUs against NCBI and ***Mare-MAGE*** databases with identical parameters. Only if an OTU had a similarity of more than 97% with a database entry and query around 100% it was assigned as a species. R and Excel were used to generate the figures.

## Data records

The ***Mare-MAGE*** databases are available via the ***Mare-MAGE*** interface at http://mare-mage.weebly.com/. The web interface is divided into 5 sections: Home, Databases, search, tutorials, Contact. It is possible to download datasets in ARB, FASTA and txt formats. It is also possible to download files in Qiime2 and Mothur formats. The tutorial includes the use of the ***Mare-MAGE*** databases with Qiime2. Every sub-database includes a file entitled “Search-DB.csv” which includes the raw information about the origin of every sequence.

It is also possible to have access from *figshare* [26], through the link https://doi.org/10.6084/m9.figshare.c.5410161.v1. Each of the 12S and COI folders includes 3 subfolders, the full or raw database (***Mare-MAGE-DBAll***), the 90% confidence database (***Mare-MAGE-90DB***) and the complete gene database (***Mare-MAGE-DBc***). All R and Python scripts used in this work are available in GitHub at https://github.com/kasmiyassin/Mare-MaGe-Database.

## Utility and discussion

### First database validation

The ***Mare-MAGE-12sDBAll*** database contains the mitochondrial 12S rRNA sequence information of 3,236 genera and 11,236 species of fish with sequence lengths between 92 and 18,000 Bp (Table 1). The ***Mare-MAGE-COI-DBAll*** contains the mitochondrial COI gene sequence information of 3,841 genera and more than 20,657 species of fish with sequence lengths between 92 and 17,000 Bp (Table 2).

**Table 1:** Statistics for 12sDB

Name	*Mare-MAGE-12sDBAll*	*Mare-MAGE- 12 s-90DB*	*Mare-MAGE- 12 s-DBc*
Total number of sequences	37 888	10 591	6487
Species	11,236	393	309
Genera	3236	242	204
Families	535	254	94
Lengths	92–18,000 Bp	100–1300 Bp	900–1200 Bp
Origin	ENA + NCBI	12sDBAll	12 s-90DB
Alignment	No	Yes	Yes
Database files	ARB, MySQL, Excel, FASTA	ARB, MySQL, Excel, FASTA, aln	ARB, MySQL, Excel, FASTA, aln
Taxonomy revision	Yes (FishBase.org)	According 12sDBAll	According 12sDBAll

**Table 2:** Statistics for COI-DB

Name	*Mare-MAGE-COI-DBAll*	*Mare-MAGE-COI-90DB*	*Mare-MAGE-COI-DBc*
Total number of sequences	193,445	153,704	4639
Species	20,657	4495	1304
Genera	3841	1741	446
Families	553	553	102
Lengths	100–17 000 Bp	500–1000 Bp	900–1600 Bp
Origin	ENA + NCBI	COI-DBAll	COI-90DB
Alignment	No	Yes	
Database files	ARB, MySQL, Excel, FASTA	ARB, MySQL, Excel, FASTA, aln	ARB, MySQL, Excel, FASTA, aln
Taxonomy revision	Yes (FishBase.org)	According COI-DBAll	According COI-DBAll

**Fig. 3:**
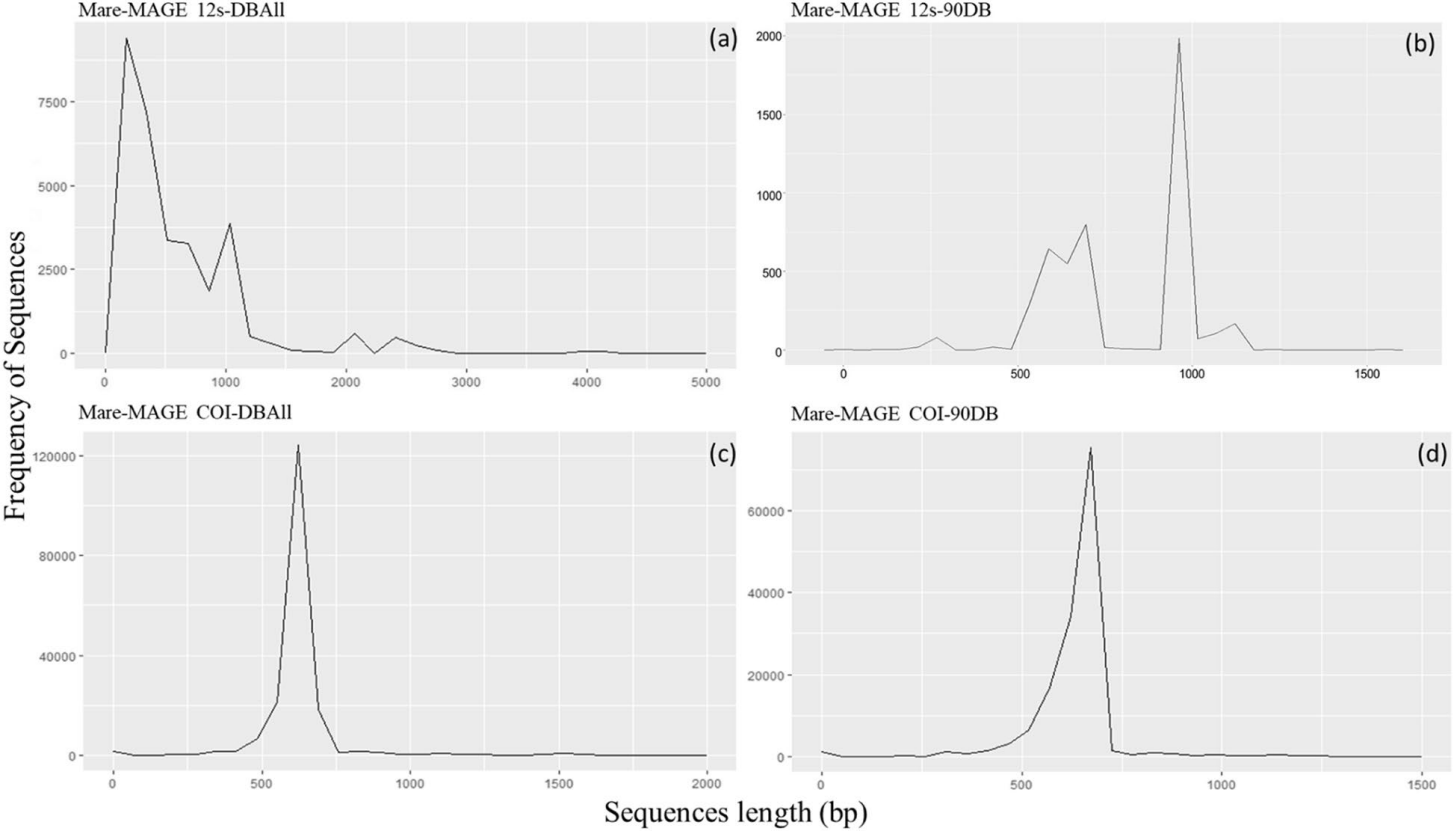
Sequence lengths distribution in ***Mare-MAGE*** Database, for 12 s rRNA and COI genes in the DB Full and 90% DB database. **a *Mare-MAGE Database 12 s-DBAll***, **b *Mare-MAGE Database 12 s-90DB***, **c *Mare-MAGE Database COI-DBAll***, **d *Mare-MAGE Database COI-90DB***. The solid line represents the sequence distribution directly after import. For ***12 s-DBAll***, most of the sequences are between 100 and 250 Bp

The ***Mare-MAGE- 12 s-90DB*** database covers 10,591 sequences (27.5% of ***Mare-MAGE- 12 s-DBAll***), of 242 genera and 393 species of fish with sequence lengths between 100 and 1,300 Bp (Table 1 and Fig. 3). More than 150,000 sequences of 1,741 genera and more than 4,400 species remain in the ***Mare-MAGE-COI-90DB*** (Table 2 and Fig. 3).

The ***Mare-MAGE- 12 s-DBc*** database contains 6,487 sequences (17.1% of ***Mare-MAGE- 12 s-DBAll***), of 204 genera and 309 species of fish with sequence lengths between 900 and 1,200 Bp (Table 1). More than 4,639 sequences of 429 genera and more than 1,304 species are included in ***Mare-MAGE-COI-DBc*** (Table 2 and Fig. 3).

The current alignment for the ***Mare-MAGE-DBAll*** is based on 37,888 and 150,000 positions for 12S and COI genes, respectively. The reasons for the large number of alignment positions are either (1) large insertions and/or (2) sequencing errors, such as additional artificial bases often found in the stretching of homopolymer sequences. As presented in section 3 (Database composition), this sub-database reflects the raw-data collected from various public databases (GenBank, NCBI, AquaGene, BOL), typically containing all 12S or COI sequences available for fish without further correction or validation, except for some taxonomic revisions according to the new nomenclature published by ITIS and FishBase. So, users need to be aware that ***Mare-MAGE DBAll*** contains some false annotations of genes or species.

While the total alignment output for all sequences of ***Mare-MAGE-90DB*** constitutes 10,591 and 6,370 positions for 12S and COI genes, respectively, for species of the same family it is restricted to ~ 2500 and ~ 2000 positions. The quality and consistency of all seed alignments in ***Mare-MAGE-90DB*** are excellent, thanks to the smaller number of alignments position. Some minor inconsistencies that we could not resolve mainly for the 12S gene in ***Mare-MAGE-90DB***, were: (1) a lack of resolution between closely related congeneric species, as for the Scombridae genus *Thunnus*, and (2) editing problems: despite manual revision, we sometimes cannot ensure that species information is provided by at least two different authors due to some misspellings of author’s names. However, these two minor inconsistencies do not significantly influence the performance and the accuracy of the two sub-databases ***Mare-MAGE-90DB*** and ***Mare-MAGE-DBc***.

### Second database validation: taxonomic assignment

**Fig. 4:**
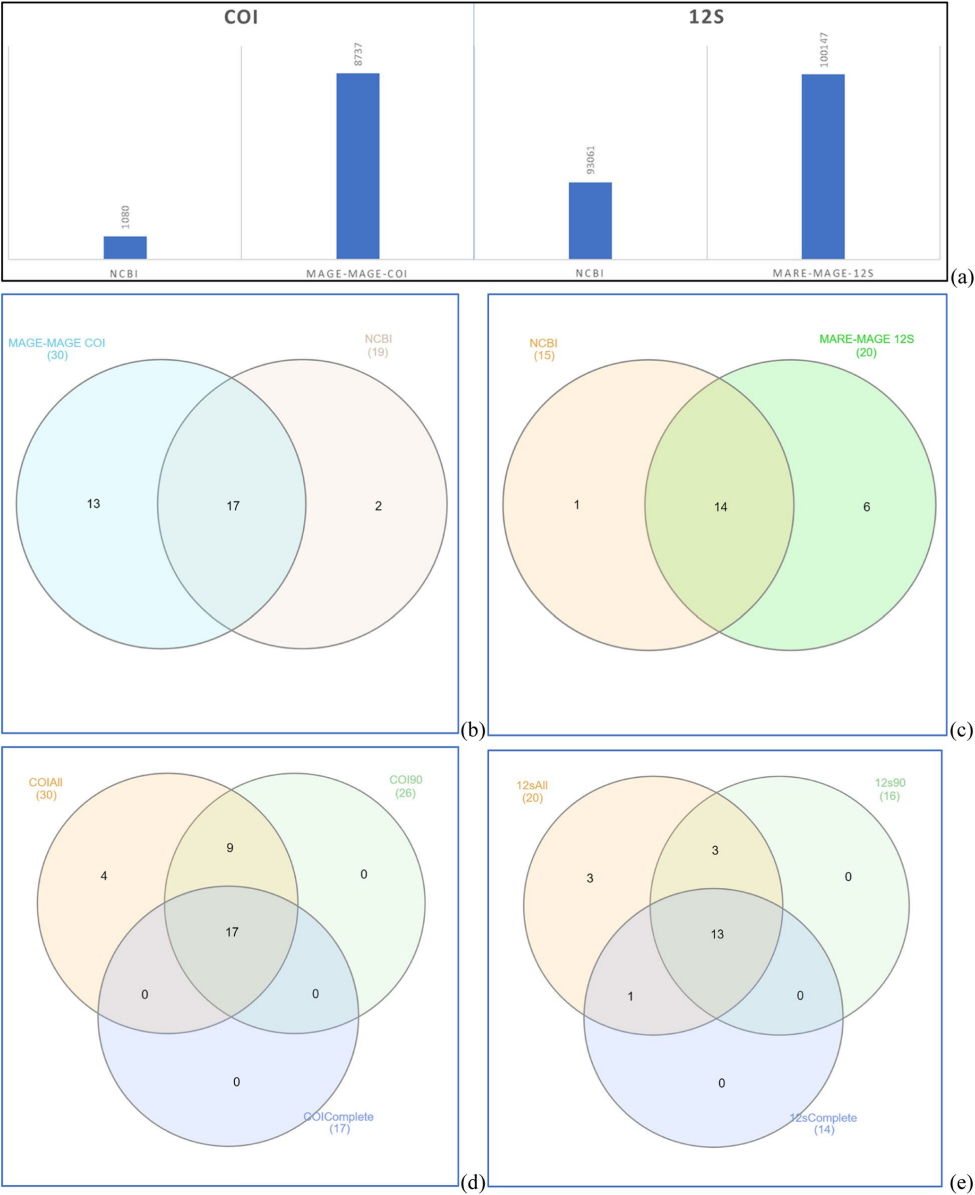
Comparison between based on OTUs assigned to a distinct species. **a** Comparison of assigned read numbers. **b** Comparison of the number of identified species using NCBI and ***MARE-MAGE*** based on the COI gene. **c** Comparison of the number identified of species using NCBI and ***MARE-MAGE*** based on the 12S rRNA gene. **d** Comparison of the number identified species using COI databases, **e** Comparison of number of species identified 12S databases

COI and 12S rRNA raw dataset analyses yielded 1,613,759, and 178,419 sequence reads, respectively, of which 104,077 merged sequence reads (58.33%) could be assembled from the 12S rRNA file, and 1,283,078 sequence reads (79.50%) from COI file. With ***Mare-MAGE*** as reference database, 96.3% of all 12S rRNA sequence reads were assigned to fish taxa with more than 97% similarity, while with NCBI only 89.4%. For COI, 8,737 sequences reads (0.68%) could be successfully assigned to fish taxa with ***Mare-MAGE*** as reference database with a similarity higher than 97%, while only 1,080 (0.08%) with NCBI (Fig. 4a). The number of fish reads assigned with ***Mare-MAGE*** was significantly higher than with NCBI (Mann–Whitney–Wilcoxon Test, *p* < 0.05).

**Fig. 5:**
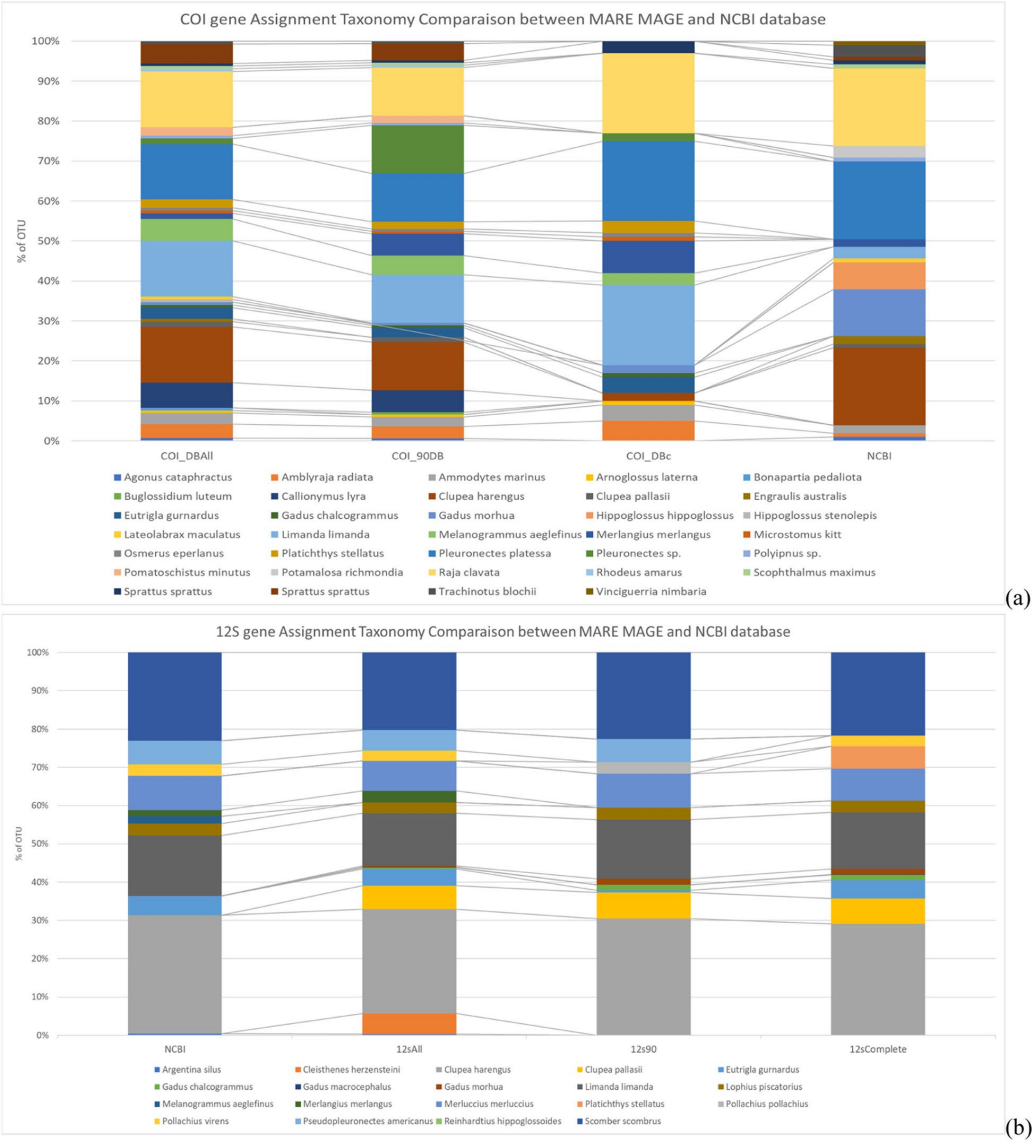
Comparison of the OTU assignment between ***Mare-MAGE*** and NCBI database based on datasets from the mitochondrial (**a**) the COI and (**b**) 12S rRNA genes

For 12S rRNA, 21 fish species out of 8 families, while for COI, 32 species out of 20 families were detected by both databases (Fig. 5). Only about 40% of the OTUs could be assigned to a distinct species with NCBI, while about 90% of the OTUs were assigned to a distinct species with ***Mare-MAGE***. The remaining OTUs were assigned either to a genus (at similarities between 97 and 92%) or to a family (at similarities between 92 and 80%). For all other datasets obtained (query coverage less than 160), 32 and 134 fish species (cartilaginous and bony fish) were recorded for 12S rRNA and COI respectively (Supporting file S1). The comparison shown in Fig. 4b-e and Tables S1 and S2 clearly shows a significant difference between ***Mare-MAGE*** versus NCBI as reference for taxonomic assignment. From the COI dataset, 13 species were solely detected with the ***Mare-MAGE*** database compared to only a single species with NCBI. For the 12S rRNA gene, 6 species were detected solely with ***Mare-MAGE*** compared to a single species with NCBI. The reason why some species were not detected by ***Mare-MAGE*** even though they were detected by NCBI is that ***Mare-MAGE*** database only covers NCBI until January 2022. An update is planned for December 2022, so any shortcomings will be regularly overcome. Despite the observed differences in the detection of OTUs between the two databases, there was also considerable overlap. For COI, 17 of the 19 species assigned by NCBI were also assigned by ***Mare-MAGE***, while for 12S rRNA, 14 of the 15 species assigned by NCBI were also present in the ***Mare-MAGE*** output. Interestingly, the number of reads assigned based on NCBI taxonomy was significantly lower than those based on ***Mare-MAGE*** for both genes (Fig. 4a).

In addition, the use of ***Mare-MAGE*** significantly improved the taxonomic assignment of fish species, by reducing the false assignment noise from microbial or viral sequences in NCBI. ***Mare-MAGE*** also includes certain species from various databases, that are still not included or synchronized with NCBI. The use of ***Mare-MAGE*** also reduced processing times, because all non-fish reads were automatically assigned as “unknown”. The ***Mare-MAGE*** database is compatible with almost all the above cited pipelines, allowing an easy and user-friendly data presentation in different graphical formats, depending on research question and pre-processing steps.

Figure 5 and Tables S1 and S2 present the percentage of reads assigned to species depending on the used reference database. The use of ***Mare-MAGE*** resulted in an increase in species number for both genes, because more OTUs were assigned to fish with a similarity above 97% and a complete coverage of the query sequenced fragment. Using NCBI for the same datasets, a considerable number of OTUs was assigned to microbial and virus at fragment lengths of less than 70 bp, negatively affecting the taxonomic assignment and rendering many fish species invisible in the output.

On the other hand, NCBI is not compatible to be used with both pipelines DADA2 and Qiime2, without extra pre-processing steps. Prior to any analysis, NCBI requires a rather complex creation of output files to reach compatibility with the pipelines. The taxonomic output is limited to the species level, preventing the possibility to get a visualization of the results at different taxonomic hierarchy levels. However, ***Mare-MAGE*** database, for both genes, was fully compatible to be uploaded into Qiime2 and DADA2 pipelines, using the command “qiime feature-classifier classify-consensus-blast” or “qiime feature-classifier classify-sklearn” for Qiime2 and “assignTaxonomy” in dada2 pipeline without any error. Another disadvantage of using NCBI on eDNA metabarcoding data is the presence of virus, bacterial and phytoplankton OTUs in the final results, which requires a post-filtering of non-fish OTUs, presuming a decent knowledge on fish taxonomy.

While non-fish OTUs (bacteria, viruses …) quite regularly occur in eDNA outputs, the availability of a database containing only fish taxa will help reducing a bias of false assignment as well as time and effort imposed on users to eliminate all non-fish sequences from the final results. In this context, the validation results clearly showed that ***Mare-MAGE*** significantly reduces the noise of non-fish OTUs compared to NCBI. Non-fish OTUs represent between 10 and 90% of the results obtained from NCBI, depending on the fish sequence diversity and the primer specificity. This noise of non-fish OTUs sometimes exceeds 100 OTUs per sample as an NCBI output, which need to be removed with considerable effort. Therefore, ***Mare-MAGE*** represents a highly reliable alternative to other public databases for taxonomic assignment of fish eDNA.

## Conclusion

In summary, the ***Mare-MAGE*** database [26] contains all 12S rRNA and COI fish sequences available at ENA and NCBI databases (last download: 21 January 2022 for 12S rRNA gene and 26 January 2022 for COI gene) with a revised and updated taxonomy based on FishBase. It contains additional sub-databases: ***Mare-MAGE-90DB*** for 90% confidence sequences belonging to a species. It is recommended to be used for fish eDNA analyses, ***Mare-MAGE-AllDB*** can be also used as *Mare-MAGE-90DB* for the fish environmental DNA analysis, but users should be careful during use, as it may contain assignment taxonomic. ***Mare-MAGE-DBc*** for sequences that cover the complete gene, it is suitable to be used for the development and validation of new primers or when the primers cover area not covered with the MiFish primer and L14912 or L14841 primers for 12S rRNA and COI genes, respectively.

***Mare-MAGE*** database is compatible for use with various pipelines, like Vsearch, Qiime2, Mothur, ObiTools. In particular allows the new database the export of ARB and FASTA files of sequences and txt files of names of taxa. Moreover, point-and-click access allows for a simple and fast import and export of sequence records and searching and mapping is provided via Qiime2.

***Mare-MAGE*** [26] will be updated yearly in December, with the second update scheduled for 2022. In the same context of improving the database we in the future aim to include also the NADH-ubiquinone oxidoreductase (ND) gene family, mainly NADH-ubiquinone oxidoreductase chain 5 (ND5).

## Supplementary Information

**Additional file 1: Table S1.** Comparison table of fish taxonomic assignments and reads between *Mare-MAGE* and NCBI, and the 3 sub-databases for the COI gene. **Table S2.** Comparison table of number of Fish taxonomic assignments and reads between *Mare-MAGE* and NCBI, and the 3 sub-databases for the 12s rRNA gene.

## Data Availability

All codes and scripts used in this work are available in Github: https://github.com/kasmiyassin/Mare-MaGe-Database. Code Unix XML NCBI was used to download the raw data under xml format. The codes R XML to csv and Python XML to csv were used to transfer XML to MS Excel format. Code R sequences from NCBI by R were used to download sequences and also the raw data species. DBAll analysis.R were used for treatment of DBAll data. R code for 90DB were used for 90DB database.
