# Supplementary material for: Mare-MAGE curated reference database of fish mitochondrial genes
Source: BMC Genom Data. 2023 Mar 17;24:18. doi: 10.1186/s12863-023-01119-4 (PMC10024356; doi:10.1186/s12863-023-01119-4)
Supplement: Supplementary file 1 — Additional file 1: Table S1. Comparison table of fish taxonomic assignments and reads between Mare-MAGE and NCBI, and the 3 sub-databases for the COI gene. Table S2. Comparison table of number of Fish taxonomic assignments and reads between Mare-MAGE and NCBI, and the 3 sub-databases for the 12s rRNA gene. [file 12863_2023_1119_MOESM1_ESM.docx]

**Supplementary Data**

**Table S1:** Comparison table of fish taxonomic assignments and reads between ***Mare-MAGE*** and NCBI, and the 3 sub-databases for the COI gene.

| **Species** | **COI_DBAll** | **COI_90DB** | **COI_DBc** | **NCBI** |
| --- | --- | --- | --- | --- |
| *Agonus cataphractus* | 11 | 10 | 0 | 10 |
| *Amblyraja radiata* | 50 | 50 | 25 | 15 |
| *Ammodytes marinus* | 4 | 4 | 4 | 2 |
| *Arnoglossus laterna* | 12 | 12 | 12 | 0 |
| *Bonapartia pedaliota* | 15 | 0 | 0 | 0 |
| *Buglossidium luteum* | 0 | 19 | 0 | 0 |
| *Callionymus lyra* | 54 | 32 | 0 | 0 |
| *Clupea harengus* | 1236 | 162 | 2 | 20 |
| *Clupea pallasii* | 21 | 18 | 0 | 19 |
| *Engraulis australis* | 10 | 0 | 0 | 2 |
| *Eutrigla gurnardus* | 4 | 4 | 4 | 0 |
| *Gadus chalcogrammus* | 6 | 1 | 1 | 0 |
| *Gadus morhua* | 10 | 1 | 2 | 12 |
| *Hippoglossus hippoglossus* | 0 | 0 | 0 | 7 |
| *Hippoglossus stenolepis* | 10 | 1 | 0 | 0 |
| *Lateolabrax maculatus* | 10 | 0 | 0 | 15 |
| *Limanda limanda* | 2680 | 260 | 250 | 30 |
| *Melanogrammus aeglefinus* | 120 | 23 | 16 | 0 |
| *Merlangius merlangus* | 280 | 125 | 10 | 210 |
| *Microstomus kitt* | 12 | 12 | 10 | 0 |
| *Osmerus eperlanus* | 15 | 16 | 12 | 0 |
| *Platichthys stellatus* | 3 | 3 | 3 | 0 |
| *Pleuronectes platessa* | 20 | 20 | 20 | 20 |
| *Pleuronectes sp.* | 2 | 20 | 2 | 0 |
| *Polyipnus sp.* | 10 | 10 | 0 | 10 |
| *Pomatoschistus minutus* | 30 | 30 | 0 | 0 |
| *Potamalosa richmondia* | 0 | 0 | 0 | 3 |
| *Raja clavata* | 20 | 20 | 20 | 20 |
| *Rhodeus amarus* | 10 | 10 | 0 | 0 |
| *Scophthalmus maximus* | 10 | 10 | 0 | 10 |
| *Sprattus sp.* | 10 | 10 | 30 | 10 |
| *Sprattus sprattus* | 70 | 70 | 0 | 10 |
| *Trachinotus blochii* | 10 | 10 | 0 | 3 |
| *Vinciguerria nimbaria* | 0 | 0 | 0 | 10 |

**Table S2:** Comparison table of number of Fish taxonomic assignments and reads between ***Mare-MAGE*** and NCBI, and the 3 sub-databases for the 12s rRNA gene.

| **Species** | **NCBI** | **12sAllDB** | **12s90DB** | **12sDBc** |
| --- | --- | --- | --- | --- |
| *Argentina silus* | 325 | 325 | 0 | 0 |
| *Cleisthenes herzensteini* | 0 | 5307 | 0 | 0 |
| *Clupea harengus* | 26846 | 26839 | 26844 | 26845 |
| *Clupea pallasii* | 16 | 6090 | 6090 | 6093 |
| *Eutrigla gurnardus* | 4407 | 4407 | 407 | 4407 |
| *Gadus chalcogrammus* | 0 | 320 | 1320 | 1320 |
| *Gadus macrocephalus* | 0 | 61 | 61 | 61 |
| *Gadus morhua* | 0 | 357 | 1357 | 1357 |
| *Limanda limanda* | 13617 | 13618 | 13616 | 13618 |
| *Lophius piscatorius* | 2784 | 2784 | 2784 | 2784 |
| *Melanogrammus aeglefinus* | 1585 | 0 | 0 | 0 |
| *Merlangius merlangus* | 1372 | 2957 | 0 | 0 |
| *Merluccius merluccius* | 7784 | 7784 | 7784 | 7784 |
| *Platichthys stellatus* | 0 | 30 | 20 | 5301 |
| *Pollachius pollachius* | 12 | 10 | 2652 | 0 |
| *Pollachius virens* | 2649 | 2650 | 0 | 2652 |
| *Pseudopleuronectes americanus* | 5309 | 5310 | 5310 | 0 |
| *Reinhardtius hippoglossoides* | 0 | 10 | 10 | 10 |
| *Scomber scombrus* | 19990 | 19990 | 19990 | 19990 |
| *Sprattus sprattus* | 6074 | 70 | 70 | 80 |
| *Trisopterus luscus* | 301 | 1336 | 1336 | 0 |
